# Administration of two probiotic strains during early childhood does not affect the endogenous gut microbiota composition despite probiotic proliferation

**DOI:** 10.1186/s12866-017-1090-7

**Published:** 2017-08-17

**Authors:** Martin Frederik Laursen, Rikke Pilmann Laursen, Anni Larnkjær, Kim F. Michaelsen, Martin Iain Bahl, Tine Rask Licht

**Affiliations:** 1 National Food Institute, Technical University of Denmark, Kemitorvet, DK-2800 Lyngby, Denmark; 2 Department of Nutrition, Exercise and Sports, University of Copenhagen, Rolighedsvej 26, DK-1958 Frederiksberg C, Denmark

**Keywords:** Probiotic intervention, LGG®, BB-12®, Early life, Gut microbiota

## Abstract

**Background:**

Probiotics are increasingly applied to prevent and treat a range of infectious, immune related and gastrointestinal diseases. Despite this, the mechanisms behind the putative effects of probiotics are poorly understood. One of the suggested modes of probiotic action is modulation of the endogenous gut microbiota, however probiotic intervention studies in adults have failed to show significant effects on gut microbiota composition. The gut microbiota of young children is known to be unstable and more responsive to external factors than that of adults. Therefore, potential effects of probiotic intervention on gut microbiota may be easier detectable in early life. We thus investigated the effects of a 6 month placebo-controlled probiotic intervention with *Bifidobacterium animalis* subsp. *lactis* (BB-12®) and *Lactobacillus rhamnosus* (LGG®) on gut microbiota composition and diversity in more than 200 Danish infants (*N* = 290 enrolled; *N* = 201 all samples analyzed), as assessed by 16S rRNA amplicon sequencing. Further, we evaluated probiotic presence and proliferation by use of specific quantitative polymerase chain reaction (qPCR).

**Results:**

Probiotic administration did not significantly alter gut microbiota community structure or diversity as compared to placebo. The probiotic strains were detected in 91.3% of the fecal samples from children receiving probiotics and in 1% of the placebo treated children. Baseline gut microbiota was not found to predict the ability of probiotics to establish in the gut after the 6 month intervention. Within the probiotics group, proliferation of the strains LGG® and BB-12® in the gut was detected in 44.7% and 83.5% of the participants, respectively. A sub-analysis of the gut microbiota including only individuals with detected growth of the probiotics LGG® or BB-12® and comparing these to placebo revealed no differences in community structure or diversity.

**Conclusion:**

Six months of probiotic administration during early life did not change gut microbiota community structure or diversity, despite active proliferation of the administered probiotic strains. Therefore, alteration of the healthy infant gut microbiota is not likely to be a prominent mechanism by which these specific probiotics works to exert beneficial effects on host health.

**Trial registration:**

NCT02180581. Registered 30 June 2014.

**Electronic supplementary material:**

The online version of this article (doi:10.1186/s12866-017-1090-7) contains supplementary material, which is available to authorized users.

## Background

Probiotics are defined as “live microorganisms which, when administered in adequate amounts, confer a health benefit on the host” [1]. Several mechanisms behind probiotic effects have been suggested, including modulation of the endogenous gut microbiota [2–4]. However, a recent meta-analysis of seven probiotic intervention studies showed that probiotic administration did not affect the adult gut microbiota as determined by analysis of fecal samples [5]. Still, in infants and children, the microbiota is known to be much more variable [6] and easier to manipulate [7] than seen for adults. Thus, if alteration of the gut microbiota composition is a mechanism by which probiotics exert their beneficial effect, this would be expected to be evident in early life interventions. In the present study, we have analyzed the fecal microbiota of healthy infants at the age of starting daycare (age 8–13 months), sampled before and after a 6 months intervention with a single daily dose of *Lactobacillus rhamnosus* (LGG**®**) and *Bifidobacterium animalis* subsp. *lactis* (BB-12**®**) or placebo. Additionally, in contrast to previous studies, we specifically assessed the presence and proliferation of the probiotic strains in the gut, and evaluated the effect on the endogenous microbial composition and diversity based on probiotic propagation.

## Methods

### Study participants

Infants, aged 8–14 months and starting daycare within 12 weeks after intervention start, were included in the ProbiComp study [8], a double blinded randomized placebo-controlled intervention. BB-12® and LGG® are registered trademarks of Chr. Hansen A/S who kindly provided the study products. During the 6 months intervention period participants were randomized to receive either a combination of the two probiotics BB-12® and LGG® at a dose of 10^9^ colony-forming units (CFU) each, or a placebo (maltodextrin), during the winter season. Participants were instructed not to consume food products and supplements containing probiotics 2 weeks prior to and during the intervention period. The study spanned two consecutive winter seasons and included a total of 290 infants (145 for each season). Study population characteristics (Table 1) were obtained through parental interviews.

**Table 1:** Characteristics of study participants with gut microbiota data completing the study (*n* = 201)

Parameter	Probiotics group (*n* = 103)	Placebo group (*n* = 98)	*P*-value
Gender			
Girls (%)	45.6	46.9	0.888^a^
Boys (%)	54.4	53.1	
Mode of delivery			
C-section (%)	23.3	15.3	0.159^a^
Vaginal (%)	76.7	84.7	
Older siblings			
Siblings (%)	48.5	43.9	0.572^a^
No siblings (%)	51.5	56.1	
Introduction of selected foods			
Cow’s milk (median months, IQR)	8.0 (7.0–12.0)	9.0 (6.0–11.0)	0.947^b^
Meat (median months, IQR)	6.0 (6.0–7.0)	6.0 (6.0–7.0)	0.242^b^
Fish (median months, IQR)	6.0 (6.0–7.0)	6.0 (6.0–7.0)	0.544^b^
Breastfeeding prevalence			
1st visit (%)	54.4	46.9	0.325^a^
2nd visit (%)	13.6	9.2	0.380^a^
Age			
1st visit (median months, IQR)	10.0 (9.5–10.4)	9.8 (9.5–10.4)	0.462^b^
Start daycare (median months, IQR)	10.4 (9.9–11.2)	10.3 (9.9–11.1)	0.309^b^
2nd visit (median months, IQR)	16.1 (15.6–16.5)	16.0 (15.6–16.4)	0.510^b^
Compliance			
2nd visit (median % of consumed sticks, IQR)	97 (93–99)	98 (94–100)	0.417^b^

^a^Fishers exact test

^b^Mann Whitney test

### Fecal samples, DNA extraction and PCR amplification of theV3 region of the 16S rRNA gene

Fecal samples obtained before (*N* = 265, age 8–13 months) and after (*N* = 210, age 14–19 months) the intervention were freshly delivered on the morning of the visit or had been stored in the participant’s home, either in the freezer (−18 °C) or in the fridge (4 °C) for maximally 24 h before delivery to the University of Copenhagen, Department of Nutrition, Exercise and Sports, where they were stored at −80 °C until DNA extraction. Extraction was done in random order, but the two samples obtained from the same child were always processed together (*N* = 16–20 samples/extraction round). 250 mg feces was applied and treated according to the protocol provided by the manufacturer (PowerLyzer® PowerSoil® DNA isolation kit, MoBio 12,855–100) with minor modifications: Bead beating was performed at 30 cycles/s for 10 min (Retsch MM 300 mixer mill) and the initial centrifugation steps were performed at 10,000 x g for 3 min, as recommended for clay matter. DNA quantity and quality was measured by NanoDrop® 1000 (ThermoFisher Scientific), yielding on average 85.0 ± 47.1 ng/μl DNA with A260/A280 = 1.89 ± 0.10 and A260/A230 = 1.71 ± 0.39. The PCR amplification of the V3-region of the 16S rRNA gene was performed with 5 ng community DNA as template, using 0.2 μl Phusion High-Fidelity DNA polymerase (Fisher Scientific, F-553 L), 4 μl HF-buffer, 0.4 μl dNTP (10 mM of each base), 1 μM forward primer (PBU 5′-A-adapter-TCAG-barcode-CCTACGGGAGGCAGCAG-3′) and 1 μM reverse primer (PBR 5′-trP1-adapter-ATTACCGCGGCTGCTGG-3′) in 20 μl total reaction volume. Both primers included sequencing adaptors and the forward primer additionally a unique 10–12 bp barcode (Ion Xpress™ Barcode Adapters). PCR amplification included 30s at 98 °C, 24 cycles of 15 s at 98 °C and 30s at 72 °C, followed by 5 min at 72 °C. PCR products were purified by use of HighPrep™ PCR Magnetic Beads (MAGBIO®, AC-60005) with the 96-well magnet stand (MAGBIO®, MyMag 96), according to the manufacturers recommendations. DNA quantity was measured using Qubit® dsDNA HS assay (Invitrogen™, Q32851) and a total of 456 samples contained sufficient DNA for sequencing. Samples were pooled to obtain equimolar libraries containing up to 86 samples (randomized across treatment groups and age) in each library.

### DNA sequencing and data handling

Sequencing of the 16S rRNA amplicon libraries was performed using the Ion OneTouch™ and Ion PGM platform with a 318-Chip v2, generating 4–5 million reads per chip with a median length of 180 bp. Sequencing data were imported into CLC Genomic Workbench (version 8.5. CLC bio, Qiagen, Aarhus, DK) and using the NGS core tools, reads were assessed for quality (*QC report*), de-multiplexed according to barcode (*Demultiplexing*) and trimmed (*Trim sequences*) to remove barcodes and PCR primers (minimum alignment score 17/17, discard when both primers were not found) and to discard reads below 125 bp and above 180 bp. Quality filtering (−fastq_filter, maxee 1.0), dereplication (−derep_fulllength), OTU clustering (−cluster_otus, minsize 4), chimera filtering (−uchime_ref, RDP_gold database), mapping of reads to OTUs (−usearch_global, id 97%) and generation of OTU table (python, uc2otutab.py) was done according to the UPARSE pipeline [9], generating a total of 1096 non-chimeric OTUs (Operational Taxonomic Units). In QIIME [10], the OTU table was filtered (filter_otus_from_otu_table.py/filter_fasta.py) to exclude OTUs with average relative abundance below 0.005% of the total community, resulting in 330 OTUs. Taxonomy was assigned (assign_taxonomy.py), using the rdp classifier with confidence threshold 0.5 (recommended for sequences shorter than 250 bp [11]) and the GreenGenes database v. 13.8 [12]. In addition, the taxonomy of selected OTUs was confirmed/disconfirmed by BLAST [13] search against the 16S rRNA sequence database. Based on PyNAST alignment of representative OTU sequences (align_seqs.py, filter_alignment.py, default settings), including an archaea (*Methanosarcina*) full length 16S rRNA gene sequence as outgroup for rooting, a phylogenetic tree was created with FastTree (make_phylogeny.py, default settings) and re-rooted to the outgroup, which was subsequently pruned, using Dendroscope v3.5.7 [14]. Taxon abundances (average classification: Phylum: 100%, Family: 99.9%, Genus: 91.8%) and alpha diversity (Shannon index, Observed OTUs, Pielou’s evenness index) and beta diversity (UniFrac distances) were calculated in QIIME (summarize_taxa.py, alpha_rarefaction.py, beta_diversity_through_plots.py), with the sequencing depth rarefied to 10,000 sequences per sample for diversity analysis. OTU abundances were estimated by total sum scaling.

### Detection of the probiotic strains BB-12® and LGG®

#### Primer design, PCR and gel electrophoresis

Primers specific for the probiotic strains were designed using the primer3 online software tool (http://bioinfo.ut.ee/primer3-0.4.0/). Selection of primers for the LGG® strain were based on a phage-related gene target sequence [15] previously reported to be strain specific [16], whereas primers for the BB-12® strain were selected within the CRISPR-Cas system [17], specifically the Type I-U CRISPR-Cas7 gene in BB-12® (for primer sequences see Additional file 1: Table S1). Each PCR reaction contained 14.92 μl DNAse-free water, 2 μl 10X Accuprime PCR buffer II, 1 μl forward primer (0.5 μM final conc.), 1 μl reverse primer (final conc. 0.5 μM), 0.08 μl AccuPrime Taq Polymerase (Invitrogen, 12,346–086) and 1 μl template DNA (1 ng/ul) in a final volume of 20 ul. The PCR program (Veriti Thermal Cycler, Applied Biosystems™, 4,452,300) included 2 min at 94 °C, 35 cycles of 20 s at 94 °C, 20 s at 65 °C and 30 s at 68 °C, followed by a final extension for 5 min at 68 °C. The PCR products were separated on a 1.5% agarose gel with 1:10,000 volume SYBR safe (Bio-Rad) for 1 h at 100 V before imaging (Molecular Imager® GelDoc™ XR System).

#### Primer validation and probiotic quantification

To validate primer specificity, a selection of *Lactobacillus* and *Bifidobacterium* strains were cultivated on MRS (de Man, Rogosa and Sharpe, [18]) or BSM (Sigma, 88,517) agar at 37 °C under anaerobic conditions for 72 h (Additional file 1: Table S2). Genomic DNA was extracted (PowerLyzer® PowerSoil® DNA isolation kit, MoBio 12,855–100) from colony material according to the protocol provided by the manufacturer and diluted to 1 ng/μl and used for input in the PCR. For spiking fecal samples with known quantities of the strains LGG® and BB-12®, single colonies of these were propagated in 10 ml MRS medium under anaerobic conditions for 48 h at 37 °C, centrifuged at 10,000×g for 5 min at 4 °C, re-suspended in 1 ml maximum recovery diluent (MRD, Oxoid CM0733), and 10-fold serially diluted in MRD. One hundred μl of each dilution (10^0^–10^−7^) was spiked into 100 mg LGG®/BB-12® negative baseline fecal samples (based on specific qPCR of the probiotic strains) and also plated in duplicates (dilutions 10^−4^–10^−9^) on MRS plates and incubated anaerobically at 37 °C for 24 h before CFU counting. Community DNA was extracted (PowerLyzer® PowerSoil® DNA isolation kit, MoBio 12,855–100) from each spiked samples plus none-spiked controls.

#### Quantitative PCR

Each quantitative PCR (qPCR) reaction contained 5 μl PCR-grade water, 1 μl forward primer (final conc. 0.5 μM), 1 μl reverse primer (final conc. 0.5 μM), 10 μl SYBR Green I Master 2X (LightCycler® 480 SYBR Green I Master, Roche, 04887352001) and 2 μl template DNA, in a 20 μl total volume. Template DNA was either 10-fold serial dilutions of 1 ng/μl DNA extracted from pure cultures of LGG® or BB-12®, community DNA isolated from fecal samples (LGG®/BB-12® negative) spiked with 10-fold serial dilutions of LGG® or BB-12® cultures, or community DNA (5 ng/μl) extracted from fecal samples obtained during the intervention. Triplicate samples were run on the qPCR instrument (LightCycler® 480 Instrument II, Roche, 05015243001) and the program included 5 min pre-incubation at 95 °C, followed by 45 cycles with 10 s at 95 °C, 15 s at 65 °C and 15 s at 72 °C. A melting curve analysis was subsequently performed with 5 min at 95 °C, 1 min at 65 °C and continuous temperature increase (ramp rate 0.11 °C/s) until 98 °C. Data were analyzed with the LightCycler® 480 Software (v 1.5) and levels of the strains LGG® and BB-12® in samples were quantified based on standard curves generated from extracted community DNA of fecal samples spiked with 10-fold serial diluted DNA extracted from pure cultures of LGG® or BB-12®. Samples were regarded LGG® and/or BB-12® positive when at least 2 of the 3 replicates were positive, the melting curves were consistent with that of pure cultures of the respective strains, and C_t_ values were within the detection range achieved for the 10-fold dilution series standard-curves.

### Statistics

Statistics were performed with the GraphPad Prism software (v. 7.0, GraphPad Software Inc., La Jolla, CA), using the Spearman’s rank correlation, Mann-Whitney test and Fisher’s exact test or by use of specific scripts for analyzing sequencing data as implemented in QIIME v. 1.9 [10]. For comparison of beta diversity the compare_categories.py (adonis, permutations = 999) script was used and for comparison of microbial composition at different taxonomical levels the group_significance.py (mann_whitney_u, Benjamini-Hochberg False Discovery Rate (FDR) correction [19], threshold 0.05) script was applied. Alpha diversity measures were compared between groups using the Mann-Whitney test of the average of 10 iterated values obtained following rarefying to 10,000 reads per sample.

## Results

### Study population characteristics and compliance

There were no statistically significant differences in gender, mode of delivery, age at introduction to selected foods (cow’s milk, fish and meat), breastfeeding prevalence, presence of older siblings, age at daycare start or age at samplings between the probiotics and the placebo groups, thus these parameters are unlikely to have affected our results (Table 1). Median compliance to the assigned treatments was 97% and 98% in the probiotics and placebo groups, respectively (Table 1).

### Microbiota profiling

**Fig. 1:**
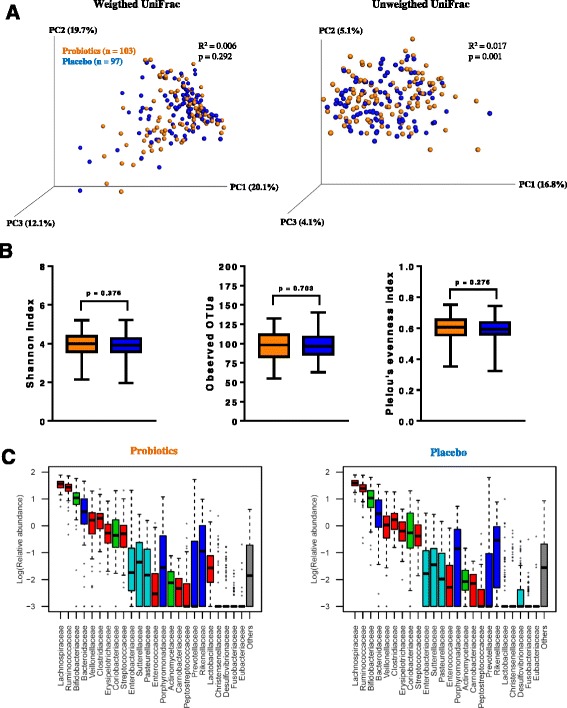
Administration of probiotics does not alter diversity and composition of the infant gut microbiota. **a** PCoA plots of weighted and unweighted UniFrac distances of the gut microbiota, **b** boxplots of gut microbial alpha diversity measures, and **c** boxplots of relative abundance of gut microbial families after probiotic (*orange*) or placebo (*blue*) intervention

Sequencing of the V3 region of the 16S rRNA genes was successful for a total of 456 fecal samples collected before (*n* = 255) and after (*n* = 201) intervention, and resulted in 11.8 million trimmed and quality filtered reads. A significant shift in alpha diversity (Shannon index, *p* < 0.0001, Mann Whitney) and beta diversity (Weighted UniFrac, R^2^ = 0.061, *p* = 0.001, Adonis) was observed during the 6 months intervention period (Additional file 1: Figure S1), which confirm a strong impact of age on gut microbiota in agreement with previous reports [20, 21]. At baseline, no differences in beta diversity (Weighted UniFrac, R^2^ = 0.010, *p* = 0.226, Adonis), or alpha diversity (Shannon index, *p* = 0.868, Pielou’s evenness index, *p* = 0.562 and Observed OTUs, *p* = 0.821) were observed between the treatment group and the placebo group (Additional file 1: Figure S2). Further, no OTUs were significantly differently abundant between the two groups after FDR correction (Additional file 1: Table S3). After the intervention, Principle Coordinate Analysis (PCoA) of both weighted and unweighted UniFrac distances did not result in visible separation of samples according to treatment group (Fig. 1a), although Adonis testing revealed a significant difference between the unweighted distances caused by the specific presence of the probiotics as described in the next section. Estimates of alpha diversity did not differ between treatment groups (Fig. 1b). Only two OTUs were found to be significantly differently abundant following FDR correction (Table 2). These were designated OTU_17 and OTU_50, and were assigned to *Bifidobacterium pseudolongum* and *Lactobacillus zeae* respectively by the GreenGenes classifier. However, a BLAST search against the 16S rRNA gene database revealed that they had 100% homology to the V3 regions of the two probiotic strains included in the intervention, namely *B. animalis* subsp. *lactis* and *L. rhamnosus* strains*,* respectively (Table 2). Testing for differential abundances at family and genus levels revealed only *Lactobacillaceae*/*Lactobacillus* as significantly higher in the probiotics group than in the placebo group (FDR adjusted *p*-value_family_ = 3.9 × 10^−14^; FDR adjusted *p*-value_genus_ = 9.1 × 10^−14^), reflecting the increased abundance of the LGG® strain in the probiotics group (Fig. 1c). Exclusion of subjects known to have consumed infant formula containing any type of probiotics at any given point during the intervention yielded similar results.

**Table 2:** OTUs significantly differentially abundant in probiotics (*n* = 103) vs. placebo (*n* = 98)

OTU ID	*P*-value	FDR *P*-value	Mean abundance (%)		NCBI BLAST hit
			Probiotics	Placebo	
OTU_17	1.45E-27	4.97E-25	2.231%	0.018%	100% *Bifidobacterium animalis* subsp. *lactis* strain YIT 4121
OTU_50	4.93E-22	8.45E-20	0.086%	0.007%	100% *Lactobacillus rhamnosus* strain NBRC 3425
OTU_6	0.013	0.870	3.049%	5.504%	100% *Eubacterium rectale* strain ATCC 33656
OTU_217	0.015	0.870	0.002%	<0.001%	99% [*Clostridium*] *lactatifermentans* strain G17
OTU_3	0.016	0.870	5.053%	7.642%	100% *Bifidobacterium pseudocatenalutum* strain B1279
OTU_290	0.016	0.870	0.009%	0.004%	97% *Butyricimonas paravirosa* strain 214–4
OTU_334	0.018	0.870	0.040%	0.001%	98% *Parasutterella excrementihominis* strain YIT 11859
OTU_25	0.037	1	0.867%	1.457%	99% *Ruminococcus bromii* strain ATCC 27255
OTU_60	0.041	1	0.184%	0.117%	100% *Barnesiella intestinihominis* strain JCM 15079
OTU_230	0.048	1	0.001%	<0.001%	94% *Ethanoligenens harbinense* strain YUAN-3

### Quantification of probiotic strains in fecal samples

Design of primers specifically targeting the probiotics LGG® and BB-12® (Additional file 1: Figure S3) allowed qPCR based quantification of these strains in the samples obtained after intervention. Strong correlations between qPCR-estimated abundances of the strain LGG® and relative abundance of OTU_50 (rho = 0.79, *p* < 0.0001) as well as between qPCR-estimated abundances of the strain BB-12® and relative abundance of OTU_17 (rho = 0.89, *p* < 0.0001), confirmed that the two OTUs represented the two probiotic strains. When these two OTUs were omitted, an unweighted UniFrac analysis with Adonis test no longer indicated any difference between the probiotic and the placebo group (R^2^ = 0.004, *p* = 0.670). By specific qPCR, both of the probiotic strains were detected in 91.3% of the infants that received probiotics and only in 1.0% of the infants receiving placebo (Table 3). The strain LGG® was detected in 91.3% whereas the strain BB-12® was detected in 95.1% of the infants treated with probiotics. However, while the LGG® strain specific primers produced an amplicon in only 2.0% of the individuals receiving placebo, the BB-12® strain primers, which are subspecies-specific, but not strain-specific (Additional file 1: Figure S3), amplified a target in 31.1% of the placebo treated infants (Table 3), suggesting an endogenous occurrence of *B. animalis* subsp. *lactis* as found in previous studies [22], or eventual prior ingestion of infant formula containing related strains. The qPCR-estimated abundances of the strain BB-12® (range 1.3 × 10^4^ to 1.5 × 10^10^ CFU/g feces) and the strain LGG® (1.5 × 10^4^ to 1.4 × 10^9^ CFU/g feces) showed high inter-individual variation. Since this variation may reflect differences in colonization resistance of the endogenous microbiota, we investigated whether selected baseline gut microbiota signatures explained the variation in the quantified amounts of the probiotic strains after intervention. However, neither alpha diversity measures nor relative abundances of individual microbial taxa at baseline correlated with the qPCR detected abundances of the administered probiotics measured at the end of the intervention (data not shown).

**Table 3:** qPCR detection of the probiotic strains in the probiotics and placebo treatment groups

	LGG® positive	BB-12® positive	LGG® & BB-12® positive
Probiotics (*n* = 103)	94/103 (91.3%)	98/103 (95.1%)	94/103 (91.3%)
Placebo (*n* = 98)	2/98 (2.0%)	32/98 (31.1%)	1/98 (1.0%)
Total (*n* = 201)	96/201 (46.8%)	130/201 (64.7%)	95/201 (47.3%)

### Estimation of probiotic proliferation in the infant gut

**Fig. 2:**
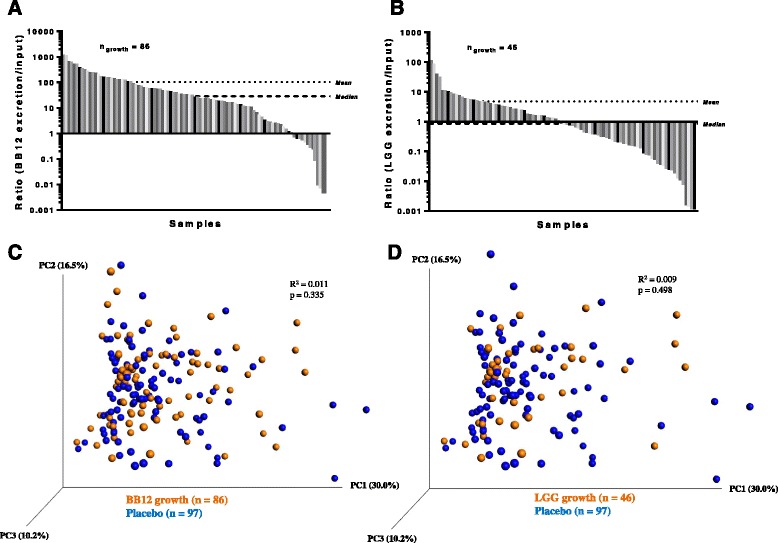
Sub-analyses of selected samples with active probiotic growth does not reveal impact on gut microbiota community structure. **a**-**b** Estimates of in situ growth of **a** BB-12**®** and **b** LGG**®** expressed as the ratio of excreted CFUs/day (excretion) to ingested CFUs/day (input) for all samples within the probiotics group. Means and medians are represented by dashed lines. **c**-**d** PCoA plots of weighted UniFrac distances of the gut microbiota in samples with detected growth of **c** BB-12**®** and **d** LGG**®** (*orange*) as compared to placebo (*blue*) intervention

In order to estimate whether or not the probiotic strains were proliferating in the gut, we determined the ratios between fecal excretion and oral intake of the two probiotic strains based on specific qPCR measurements for infants in the probiotic group. Intake of each of the probiotic strains, LGG® and BB-12®, was set as the daily dose of 10^9^ CFU. The amount of excreted probiotics was calculated as the qPCR-estimated amount of CFU/g feces multiplied with the average weight of feces reported to be excreted by healthy individuals at the given age, which is 80 g/day [23, 24]. A ratio of fecal excretion to oral input (excretion/input) above 1 was considered to indicate that probiotic growth had occurred in the gut. In this context, it should be noted that a considerable fraction of probiotics are expected not to survive gastrointestinal transit, and that even a ratio just below 1 may thus imply probiotic proliferation. For the strain BB-12®, a ratio above 1 was found in 86 of the 103 individuals (83.5%), resulting in a median excretion/intake ratio of 29. While these estimates may be affected by occurrence of other *B. animalis* subsp. *lactis* strains (which we could not differentiate from the ingested BB-12® strain by qPCR), abundance of this subspecies was on average more than 100-fold lower in the placebo group (Table 2), suggesting that the other occurring subspecies of *B. animalis subsp. lactis* made only an insignificant contribution to the growth estimates. For the LGG® strain, a ratio above 1 was observed in 46 of the 103 infants (44.7%) treated with probiotics, resulting in a median excretion/intake ratio of 0.9. Noteworthy, all infants with a LGG® ratio above 1 also had a BB-12® ratio above 1 (Fig. 2a-b). This shows that both of the probiotic strains were proliferating in the gut of at least 44.7% of the infants receiving probiotics. Comparison of the intestinal bacterial communities of the probiotics-treated infants with detected growth of either the BB-12® strain (*n* = 86) or LGG® strain (*n* = 46) to the placebo group (*n* = 97) did not reveal any differences in community structure assessed by weighted UniFrac distances (Fig. 2c-d), when OTU_17 and OTU_50 (representing the probiotics) were filtered out. Nor did we find significant differences in alpha diversity measures (Additional file 1: Figure S4). Finally, no differentially abundant OTUs were detected in this comparison (Additional file 1: Table S4 & Table S5), indicating that the composition of the endogenous microbiota was not affected despite proliferation of the probiotic strains in the infant gut.

## Discussion

The bacterial strains LGG® and BB-12® represent some of the most commonly applied commercially available probiotics, and studies of their putative effects are therefore highly relevant. We investigated the impact of a 6 months intervention with daily doses of 10^9^ CFU of these two probiotic strains on the gut microbiota of infants aged 8–13 months at onset. Community structure and diversity of the endogenous infant gut microbiota were not affected despite the fact that the gut microbiota is characterized by low stability and high responsiveness in early life [25]. Several comparable reports on probiotic treatments exist. For example, a previous intervention study with the probiotic strains BB-12®, LGG® and *L. acidophilus* LA-5® given to pregnant women from the 36th gestation week until 3-months post-partum addressed gut microbiota development in the offspring at ages 10d, 3 m, 1y and 2y [21, 26]. While significant mother-to-infant transmission of LGG® (but not of BB-12® or LA-5®) is reported, no effects on infant gut microbiota composition or diversity were found [21, 26], which is in line with our findings. Similarly, oral administration of *B. bifidum* W23, *B. lactis* W52 and *Lactococcus lactis* W58 during the last 6 weeks of pregnancy and subsequently to offspring at high risk of atopic disease during the first year of life is reported to have no effect on the endogenous infant gut microbiota composition and diversity, neither during the intervention nor at a later follow-up [27]. Also, 8 weeks daily administration of either *L. acidophilus* NCFM or *B. lactis* Bi-07 to infants aged 7–24 months and diagnosed with atopic dermatitis is reported not to change the composition and diversity of the main fecal bacterial populations as compared to a placebo group [28]. While these studies together strongly suggest that exposure to commonly applied (non-endogenous) probiotics in early life does not interfere with the natural succession and development of the infant gut microbiota, they do not consider the fact that probiotics may not colonize or grow efficiently in all individuals. In the present study, we thus further conducted sub-analyses stratifying individuals based on estimates of probiotic proliferation of the given strain after ingestion, but still found no support of the hypothesis that ingestion of the given probiotics alter the endogenous gut microbiota. Although our assessment may have underestimated the number of samples characterized by proliferation of the ingested probiotics, the selected samples with excretion/intake ratios above 1 represent the most extreme examples and would be expected to have shown an impact on gut microbiota composition if this had been affected.

## Conclusion

We show that alteration of the early life gut microbial community was not achieved by oral administration of LGG® and BB-12® despite proliferation of the ingested probiotics in the infant intestine. We thus conclude that modulation of the endogenous microbiota is unlikely to be the causal mechanism behind putative effects of these specific probiotic strains on infant health. However, this does not exclude a direct impact of the strains on the host intestinal barrier function, gut associated immune responses and/or systemic metabolic effects, which may or may not depend on probiotic proliferation.

## Additional file

Additional file 1: Table S1. Primers used to detect and quantify BB-12® and LGG®. **Table S2.** Strains used for evaluation of primer specificity. **Table S3.** OTUs with differential abundance comparing baseline samples. **Table S4.** OTUs with differential abundance comparing samples with BB-12® growth to placebo. **Table S5.** OTUs with differential abundance comparing samples with LGG® growth to placebo. **Figure S1.** Gut microbiota changes over the course of the 6 months intervention. **Figure S2.** Gut microbiota at baseline does not differ between probiotics and placebo groups. **Figure S3.** Evaluation of primer specificity for LGG® and BB-12® primers. **Figure S4.** Gut microbial alpha diversity measures are not affected by active growth of probiotics. (PDF 532 kb)

## Abbreviations

CFU: Colony forming units
FDR: False Discovery Rate
OTU: Operational Taxonomic Unit
PCoA: Principal coordinate analysis
qPCR: Quantitative polymerase chain reaction
