# Supplementary material for: Administration of two probiotic strains during early childhood does not affect the endogenous gut microbiota composition despite probiotic proliferation
Source: BMC Microbiol. 2017 Aug 17;17:175. doi: 10.1186/s12866-017-1090-7 (PMC5561568; doi:10.1186/s12866-017-1090-7)
Supplement: Additional file 1: Table S1. — Primers used to detect and quantify BB-12® and LGG®. Table S2. Strains used for evaluation of primer specificity. Table S3. OTUs with differential abundance comparing baseline samples. Table S4. OTUs with differential abundance comparing samples with BB-12® growth to placebo. Table S5. OTUs with differential abundance comparing samples with LGG® growth to placebo. Figure S1. Gut microbiota changes over the course of the 6 months intervention. Figure S2. Gut microbiota at baseline does not differ between probiotics and placebo groups. Figure S3. Evaluation of primer specificity for LGG® and BB-12® primers. Figure S4. Gut microbial alpha diversity measures are not affected by active growth of probiotics. (PDF 532 kb) [file 12866_2017_1090_MOESM1_ESM.pdf]

Additional file 1

Additional material related to:

Administration of two probiotic strains during early childhood does not affect the endogenous gut microbiota composition despite probiotic proliferation

by

Martin Frederik Laursen, Rikke Pilmann Laursen, Anni Larnkjær, Kim F. Michaelsen, Martin Iain Bahl & Tine Rask Licht.

Content:

Table S1 – Primers used to detect and quantify BB-12® and LGG®

Table S2 – Strains used for evaluation of primer specificity

Table S3 – OTUs with differential abundance comparing baseline samples

Table S4 – OTUs with differential abundance comparing samples with BB-12® growth to placebo

Table S5 – OTUs with differential abundance comparing samples with LGG® growth to placebo

Figure S1 – Gut microbiota changes over the course of the 6 months intervention

Figure S2 – Gut microbiota at baseline does not differ between probiotics and placebo groups

Figure S3 – Evaluation of primer specificity for LGG® and BB-12® primers

Figure S4 – Gut microbial alpha diversity measures are not affected by active growth of probiotics

Additional references

Table S1 – Primers used to detect and quantify BB12 and LGG

Primers	Sequence (5'-3')	Primer length	Tm (^{o}C)	GC %	Product size (bp)	Target
**BB12_F**	ACTTCAATCGCTCGGCTGATGTA	23	65.3	47.8	154	Type I-U CRIPSR-Cas7 gene [1]
**BB12_R**	TCGTAGCTCCTGCAAAATACACT	23	60.3	43.5		
**LGG_F**	GTCAACCATTGCTTGCCCTT	20	62.3	50.0	202	Phage-related gene [2]
**LGG_R**	AGAAAAGACACCGGGTTTGC	20	61.4	50.0		

Table S2 – Strains used for evaluation of primer specificity

Strain	Culture Collection
Lactobacillus rhamnosus GG	ATCC 53103
Lactobacillus rhamnosus	DSM 20021
Lactobacillus paracasei subsp. *paracasei* 85E	DSM 20006
Lactobacillus acidophilus L917	DSM 20079
Lactobacillus johnsonii VPI 7660	DSM 10533
B. animalis subsp. *lactis* UR1	DSM 10140
B. animalis subsp. *lactis* BB-12	Chr. Hansen A/S
B. animalis subsp. *lactis* BL-04	SD5219
B. breve S50	DSM 20091
B. animalis subsp. *animalis*	DSM 20104
B. pseudolongum subsp. *pseudolongum*	DSM 20099

Table S3 – OTUs with crude p-value below 0.05 for differential abundance comparing baseline samples of completers assigned to probiotics (n = 95) vs. placebo (n = 89).

OTU ID	P-value[a]	FDR P-value[b]	Mean abundance (%)		Green Genes taxonomy
			Placebo (n = 89)	Probiotics (n = 95)	
OTU_76	0.025	0.996	0.035%	0.085%	*Erysipelotrichaceae*
OTU_374	0.026	0.996	0.022%	0.053%	*Moryella*
OTU_916	0.031	0.996	0.140%	0.021%	*Bacteroides caccae*
OTU_106	0.033	0.996	0.048%	0.011%	*Sutterella*
OTU_304	0.040	0.996	0.008%	0.002%	*Erysipelotrichaceae*
OTU_800	0.043	0.996	0.003%	0.005%	*Lachnospiraceae*

[a]P-value according to Mann Whitney test.
[b]False Discovery Rate correction of the P-values.

Table S4 – OTUs with crude p-value below 0.05 for differential abundance comparing samples with BB-12® growth (n = 86) vs. placebo (n = 98).

OTU ID	P-value[a]	FDR P-value[b]	Mean abundance (%)		Green Genes taxonomy
			Placebo (n = 98)	BB-12® growth (n = 86)	
OTU_3	0.009	0.983	7.644%	4.921%	*Bifidobacterium*
OTU_334	0.021	0.983	0.001%	0.051%	*Sutterella*
OTU_60	0.022	0.983	0.117%	0.182%	*[Barnesiellaceae]*
OTU_290	0.031	0.983	0.004%	0.011%	*Butyricimonas*
OTU_6	0.034	0.983	5.504%	3.293%	*Pseudobutyrivibrio*
OTU_575	0.036	0.983	<0.001%	0.062%	*[Eubacterium] biforme*
OTU_181	0.042	0.983	0.015%	0.003%	*Lactococcus*
OTU_25	0.042	0.983	1.457%	0.949%	*Ruminococcus bromii*
OTU_313	0.048	0.983	0.173%	0.143%	*Bacteroides acidifaciens*

[a]P-value according to Mann Whitney test.
[b]False Discovery Rate correction of the P-values.

Table S5 – OTUs with crude p-value below 0.05 for differential abundance comparing samples with LGG® growth (n = 46) vs. placebo (n = 98).

OTU ID	P-value[a]	FDR P-value[b]	Mean abundance (%)		Green Genes taxonomy
			Placebo (n = 98)	LGG® growth (n = 46)	
OTU_575	0.0194	0.974	<0.001%	0.087%	*[Eubacterium] biforme*
OTU_6	0.0369	0.974	5.504%	2.464%	*Pseudobutyrivibrio*
OTU_217	0.0383	0.974	0.000%	<0.001%	*Clostridiales*
OTU_230	0.0383	0.974	0.000%	0.002%	*Ruminococcaceae*
OTU_189	0.047	0.974	0.014%	0.000%	*Megasphaera*
OTU_575	0.0194	0.974	<0.001%	0.087%	*[Eubacterium] biforme*

[a]P-value according to Mann Whitney test.
[b]False Discovery Rate correction of the P-values.

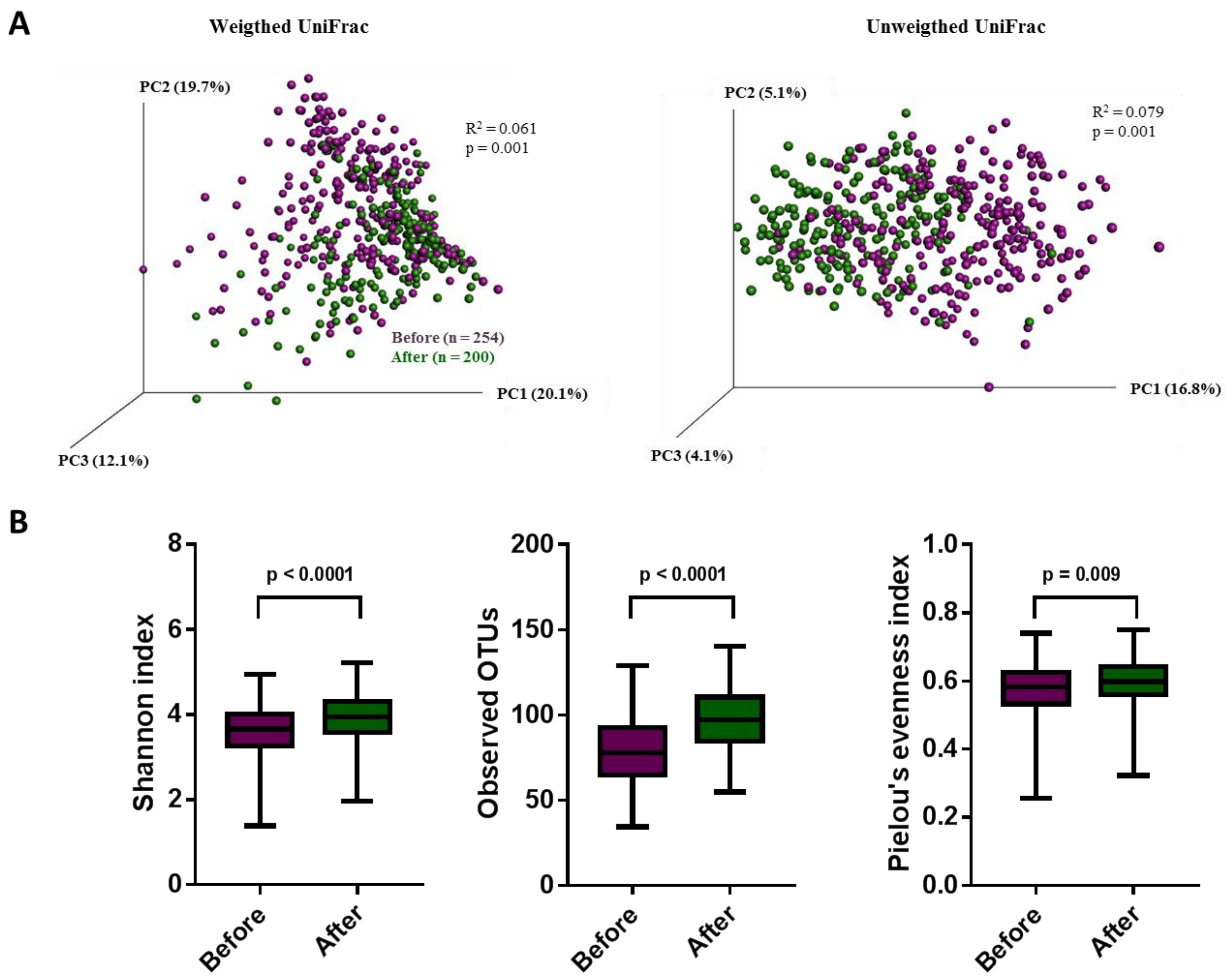

Figure S1 – Gut microbiota changes over the course of the 6 months intervention.

A) PCoA plots of weighted and unweighted UniFrac distances of the gut microbiota, B) boxplots of gut microbial alpha diversity measures before (purple) and after (green) intervention.

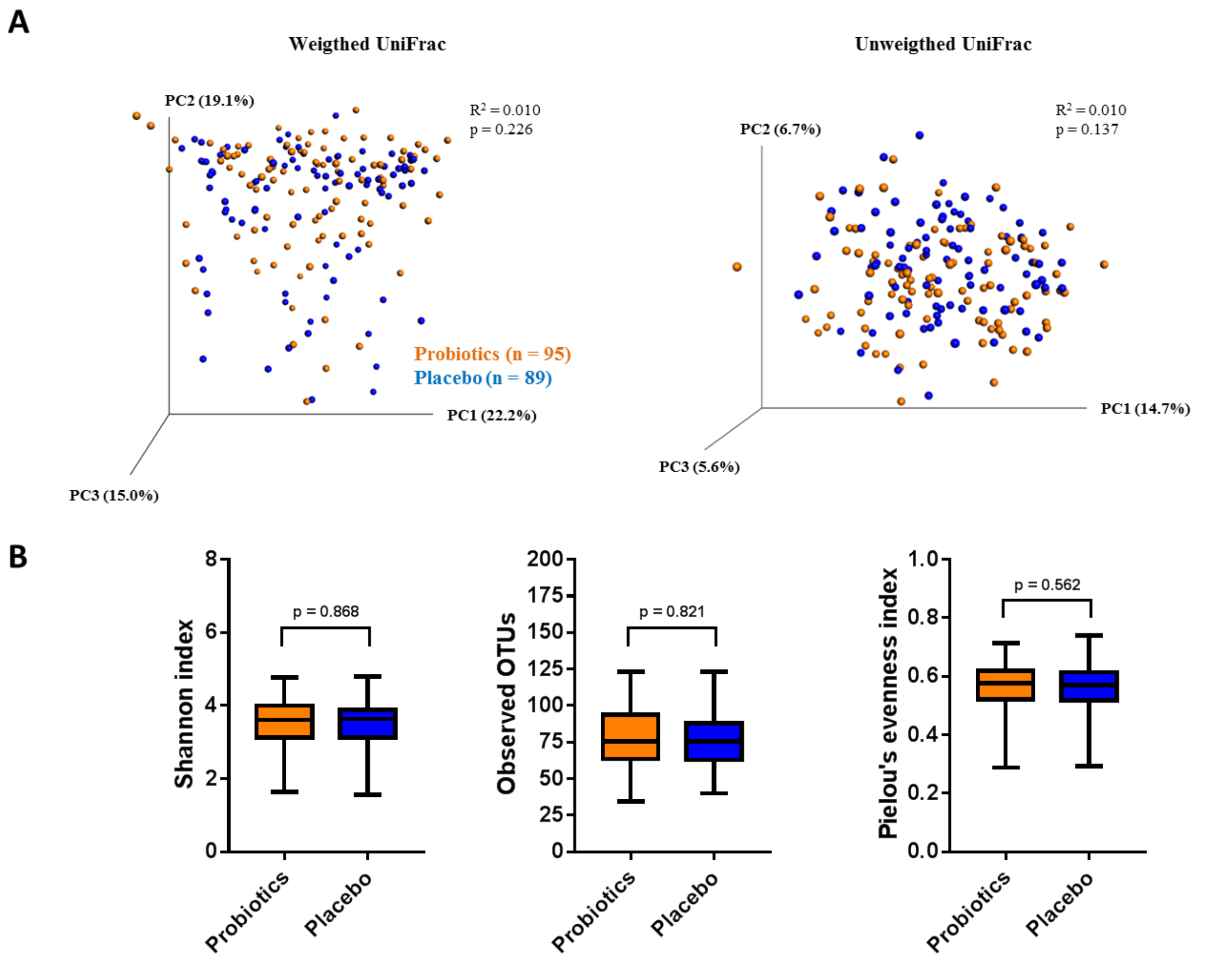

Figure S2 – Gut microbiota at baseline does not differ between probiotics and placebo groups.

A) PCoA plots of weighted and unweighted UniFrac distances of the gut microbiota, B) boxplots of gut microbial alpha diversity measures for individuals assigned to probiotics (orange) and placebo (blue) treatments.

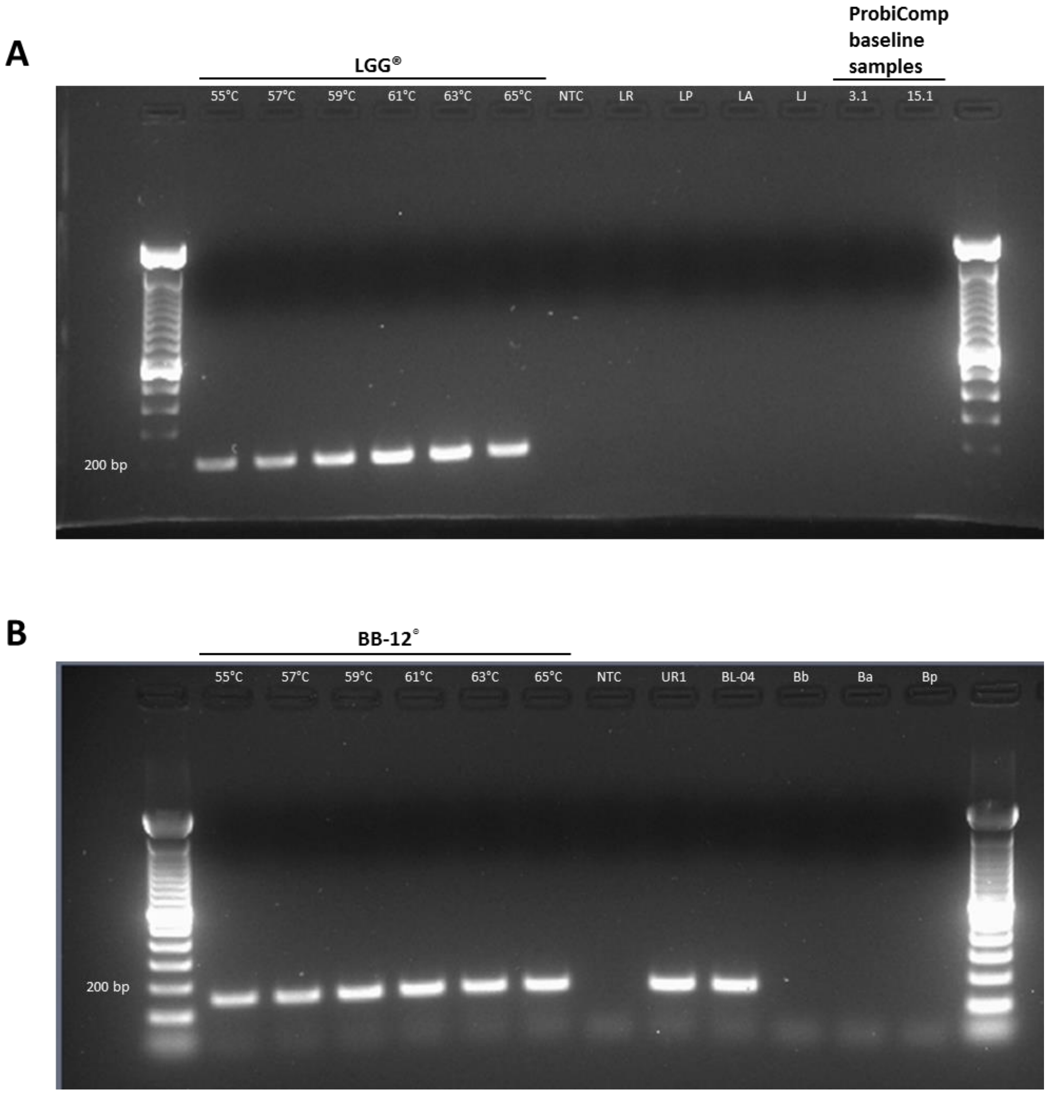

Figure S3 – Evaluation of primer specificity for LGG® and BB-12® primers.

A) UV image of a gel containing PCR products obtained with extracted DNA from *Lactobacillus rhamnosus* (LGG® at different annealing temperatures), none template control (NTC), *L. rhanmosus* DSM 20021 (LR), *L. paracasei* subsp. *paracasei* 85E DSM 20006 (LP), *L. acidophilus* L917 DSM 20079 (LA), *L. johnsonii* VPI 7660 DSM 10533 (LJ) or ProbiComp baseline samples (3.1 and 15.1) using LGG® primers. B) UV image of a gel containing PCR products obtained with extracted DNA from *B. animalis* subsp. *lactis* (BB-12® at different annealing temperatures), none template control (NTC), *B. animalis* subsp. *lactis* UR1 DSM 10140 (UR1), *B. animalis* subsp. *lactis* BL-04 SD5219 (BL-04), *B. breve* S50 DSM 20091 (Bb), *B. animalis* subsp. *animalis* DSM 20104 (Ba) or *B. pseudolongum* subsp. *pseudolongum* DSM 20099 (Bp) using BB-12® primers. A 100 bp DNA ladder (Invitrogen, 15628019) is included in the first and last wells for comparison of basepair size.

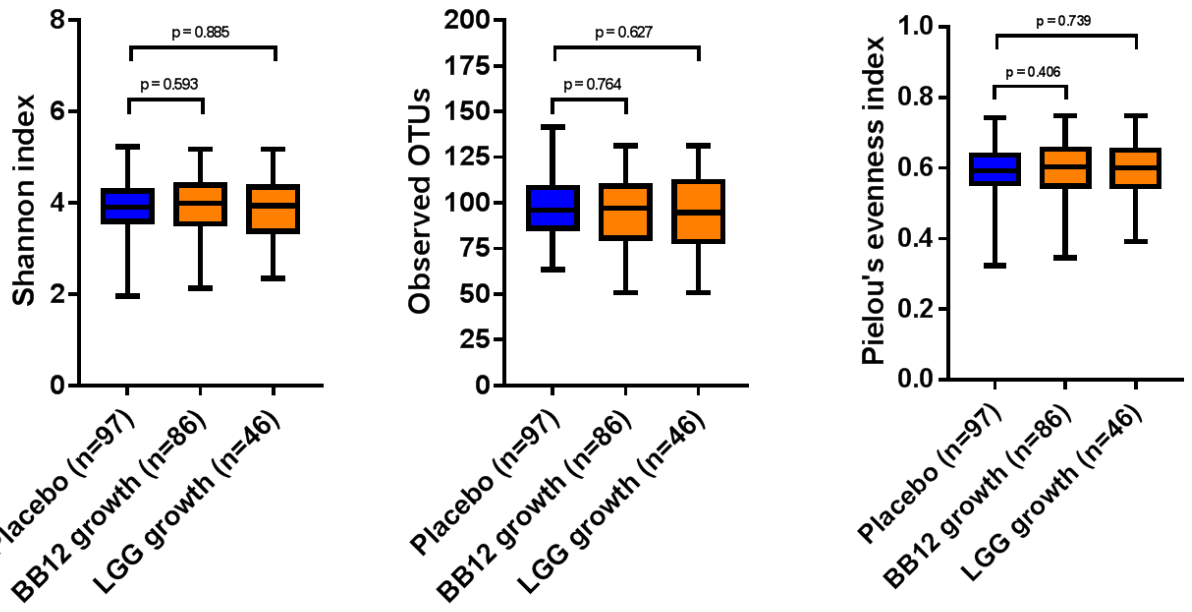

Figure S4 – Gut microbial alpha diversity measures are not affected by active growth of probiotics. Boxplots of gut microbial alpha diversity measures for individuals with detected growth of BB-12® (n = 86) or LGG® (n = 46) as compared to placebo (n = 97).

Additional References

1. Briner AE, Lugli GA, Milani C, Duranti S, Turroni F, Gueimonde M, et al. Occurrence and Diversity of CRISPR-Cas Systems in the Genus Bifidobacterium. Riedel CU, editor. PLoS One [Internet]. Public Library of Science; 2015 [cited 2016 Jul 1];10:e0133661. Available from: http://dx.plos.org/10.1371/journal.pone.0133661

2. Endo A, Aakko J, Salminen S, Ahlroos T, Tynkkynen S, Brandt K, et al. Evaluation of strain-specific primers for identification of Lactobacillus rhamnosus GG. FEMS Microbiol. Lett. [Internet]. The Oxford University Press; 2012 [cited 2016 Jul 1];337:120–5. Available from: http://www.ncbi.nlm.nih.gov/pubmed/23003331
